# *MeNINV1*: An Alkaline/Neutral Invertase Gene of *Manihot esculenta*, Enhanced Sucrose Catabolism and Promoted Plant Vegetative Growth in Transgenic *Arabidopsis*

**DOI:** 10.3390/plants11070946

**Published:** 2022-03-31

**Authors:** Ya-Jie Wang, Xing-Hou Zhen, Yang-Jiao Zhou, Yun-Lin Wang, Jing-Yi Hou, Xin Wang, Rui-Mei Li, Jiao Liu, Xin-Wen Hu, Meng-Ting Geng, Yuan Yao, Jian-Chun Guo

**Affiliations:** 1 School of Life Sciences, Hainan University, Haikou 570228, China; wyj5501@hainanu.edu.cn (Y.-J.W.); 2021110710000044@hainanu.edu.cn (X.-H.Z.); 18071010110007@hainanu.edu.cn (Y.-J.Z.); 20095131210026@hainanu.edu.cn (J.-Y.H.); 20086000210043@hainanu.edu.cn (X.W.); huxinwen@hainanu.edu.cn (X.-W.H.); 2 Key Laboratory of Biology and Genetic Resources of Tropical Crops, Ministry of Agriculture, Institute of Tropical Bioscience and Biotechnology, Chinese Academy of Tropical Agricultural Sciences, Haikou 571101, China; 13876830997@163.com (Y.-L.W.); liruimei@itbb.org.cn (R.-M.L.); liujiao@itbb.org.cn (J.L.)

**Keywords:** cassava, alkaline/neutral invertase, MeNINV1, *Arabidopsis thaliana*, sucrose metabolism, vegetative growth

## Abstract

Alkaline/neutral invertase (A/N-INV) is an invertase that irreversibly decomposes sucrose into fructose as well as glucose and plays a role in plant growth and development, starch synthesis, abiotic stress, and other plant-life activities. Cassava is an economically important starch crop in tropical regions. During the development of cassava tuber roots, A/N-INV activity is relatively high, which indicates that it may participate in sucrose metabolism and starch synthesis. In this study, MeNINV1 was confirmed to function as invertase to catalyze sucrose decomposition in yeast. The optimal enzymatic properties of MeNINV1 were a pH of 6.5, a reaction temperature of 40 °C, and sucrose as its specific catalytic substrate. VB_6_, Zn^2+^, and Pb^2+^ at low concentrations as well as EDTA, DTT, Tris, Mg^2+^, and fructose inhibited A/N-INV enzymic activity. In cassava, the *MeNINV1* gene was mainly expressed in the fibrous roots and the tuber root phloem, and its expression decreased as the tuber root grew. MeNINV1 was confirmed to localize in chloroplasts. In *Arabidopsis*, *MeNINV1*-overexpressing *Arabidopsis* had higher A/N-INV activity, and the increased glucose, fructose, and starch content in the leaves promoted plant growth and delayed flowering time but did not change its resistance to abiotic stress. Our results provide new insights into the biological function of MeNINV1.

## 1. Introduction

Sucrose and starch are the most common nonstructural carbohydrates in higher plants [1]. Starch is the main storage form of carbohydrates, while soluble sucrose is the main transport form of photosynthetic products from source organs to sink organs (such as roots and seeds) [2]. Sucrose is a carbon and energy transfer carrier and a signaling molecule involved in many physiological processes [3]. The first step of sucrose metabolism is its breakdown into two hexoses (glucose and fructose), which is important in transporting photoassimilates because it affects storage capacity [4]. In addition, the decomposition of sucrose is important for signal transduction as sucrose and hexose can induce different signal responses and play important functions in plant growth and development, biotic and abiotic stresses, starch synthesis, osmotic regulation, and other life activities [5]. Sucrose decomposition is catalyzed by two enzymes: sucrose synthase and invertase. The decomposition of sucrose into UDP-glucose and fructose by sucrose synthase is reversible, while the hydrolysis of sucrose into glucose and fructose by invertase is irreversible [6,7]. The irreversible decomposition of sucrose by invertase determines its role in plant sucrose metabolism.

There are several isoenzymes of invertase. According to their optimal pH and their isoelectric point, invertase can be divided into alkaline/neutral invertase (A/N-Inv) and acid invertase (A-Inv) [8]. Acid invertase (pH 4.5–5.0) can be divided into vacuolar invertase and cell wall invertase according to solubility and subcellular localization. A-Inv belongs to the glycoside hydrolase family GH32 [9], which can catalyze the decomposition of sugars with β-1,2 glycosidic bonds, has a high affinity for sucrose, and can also hydrolyze raffinose and stachyose. A/N-Inv is a nonglycosylated protein belonging to the glycoside hydrolase family GH100 with an optimal pH value of 6.5–8.0 [7,9,10]. A/N-Inv can only specifically catalyze the hydrolysis of sucrose [7], but some studies have found that A/N-Inv can also catalyze the decomposition of maltose [11]. The enzyme activity of A/N-Inv is inhibited by fructose, glucose, tris-(hydroxymethyl)- aminomethane (Tris), and metal ions [11]. A/N-Inv is extremely unstable and easy to inactivate during the in vitro extraction process. Therefore, physiological function research of A/N-Inv is less abundant than that of A-Inv.

As in a few previous studies, A/N-Inv only existed in the cytoplasm. With in-depth research, it has been discovered that A/N-Inv can be divided into an α-group and a β-group according to their evolutionary relationship. The A/N-Inv of the β-group are more specific, and all of them are located in the cytoplasm, while the α-group can be divided into the α1 and α2 classes. The A/N-Inv of class α1 are mostly located in the mitochondria or chloroplasts, and the A/N-Inv of class α2 are mostly located in plastids [12,13,14]. The β-group A/N-Inv might be involved in sucrose metabolism in the nonphotosynthetic cells, regulating cell development and the synthesis of secondary metabolites [15]. The C-terminus of the β-group A/N-Inv has a conserved sequence that can bind to 14-3-3 protein. The 14-3-3 protein can phosphorylate the serine residue at this binding site under the cooperation of calcium-dependent protein kinases CPK3 and CPK21 and positively regulate the activity of invertase [16]; PIP5K9 can prevent 14-3-3 protein from interacting with A/N-Inv and thereby negatively regulating the activity of invertase [17]. The α-group A/N-Inv has no binding site for 14-3-3 protein. There may be other enzyme activity regulation mechanisms, but they are not yet known. The α-group A/N-Inv may be involved in sucrose metabolism in chloroplasts or mitochondria, or both, regulating the stability of photosynthetic processes and energy conversion and affecting starch synthesis in organelles, plant germination and flowering, the balance of reactive oxygen species, and abiotic stress [18,19,20,21].

Cassava leaves have extremely high efficiency in photosynthetic carbon assimilation and sucrose synthesis, but the starch accumulation in cassava tuber roots is far lower than the potential theoretical yield [22]. A/N-Inv activity is mainly manifested in storage roots, which indicates that it may be involved in the sucrose metabolism of cassava tuber-root development and play an important role in the accumulation of tuber-root starch [23]. There are 11 A/N-Inv gene members in cassava, among which *MeNINV1*, *6*, *7*, *8*, *9*, and *10* are classified in the α-group, and *MeNINV2*, *3*, *4*, *5*, and *nINV1* are classified in the β-group [24]. Our previous studies have found that *MeNINV1* (belonging to the α-group) and *nINV1* (belonging to the β-group) are much higher than other members during the accumulation of starch in cassava tuber roots at each stage, indicating that they might be key genes for sucrose metabolism and starch accumulation in cassava tuber roots [24]. The regulatory mechanism of β-group A/N-Inv activity has been studied, but the regulatory mechanism of α-group A/N-Inv activity is not yet fully understood [16,17]. In this study, the characteristics of *MeNINV1* of the α-group A/N-Inv were investigated; these characteristics included enzymatic properties, gene expression patterns, and protein location. Finally, *MeNINV1* has been overexpressed in *Arabidopsis* to explore its role in plant growth and development, sucrose metabolism, starch accumulation, and abiotic stress.

## 2. Results

### 2.1. Functional Analysis of MeNINV1 by Yeast Complementation

**Figure 1:**
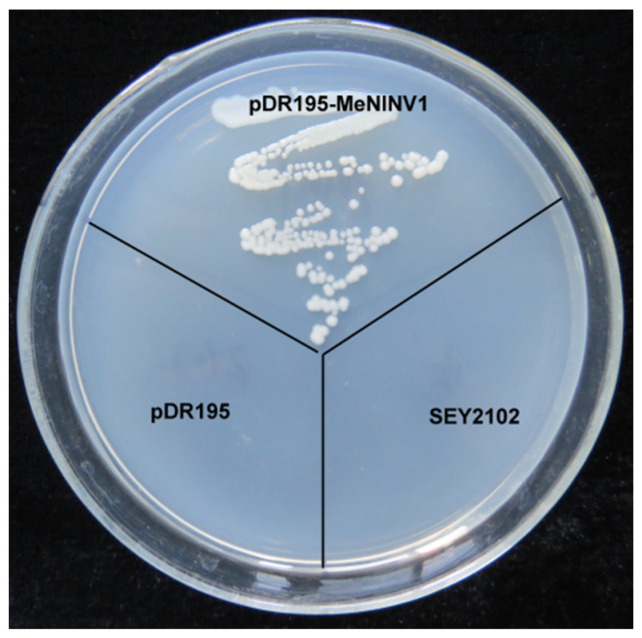
Invertase activity analysis of MeNINV1 in transgenic yeast cells. pDR195-MeNINV1: SEY2102 was transformed with pDR195-MeNINV1; pDR195: SEY2102 was transformed with pDR195; SEY2102: the invertase mutant SEY2102. Yeast cells were grown on SD medium with sucrose as the sole carbon source at 30 °C for 3 d.

Previously, 11 A/N-Inv family genes (*MeNINVs*) from the cassava genome were cloned and analyzed in our laboratory [24]. *MeNINV1* was classified into the α-group with relatively high expression levels in different tissues and stages of tuber root development. To verify whether MeNINV1 had A/N-Inv activity, the *MeNINV1* gene was cloned into the yeast-expressing vector pDR195. The recombinant plasmid was named pDR195-MeNINV1 and transformed into the invertase mutant yeast strain SEY2102. SEY2102, SEY2102 carrying pDR195, and SEY2102 carrying pDR195-MeNINV1 were streaked on Synthetic Dropout solid medium (SD medium lacking uracil) with sucrose as the sole carbon source. The results showed that SEY2102 carrying the pDR195-MeNINV1 strain grew normally, while the other two yeast strains did not (Figure 1). This result indicated that MeNINV1 can catalyze the decomposition of sucrose to provide a carbon source and energy for the growth of the invertase mutant yeast SEY2102.

### 2.2. Analysis of MeNINV1 Enzymatic Characteristics

**Figure 2:**
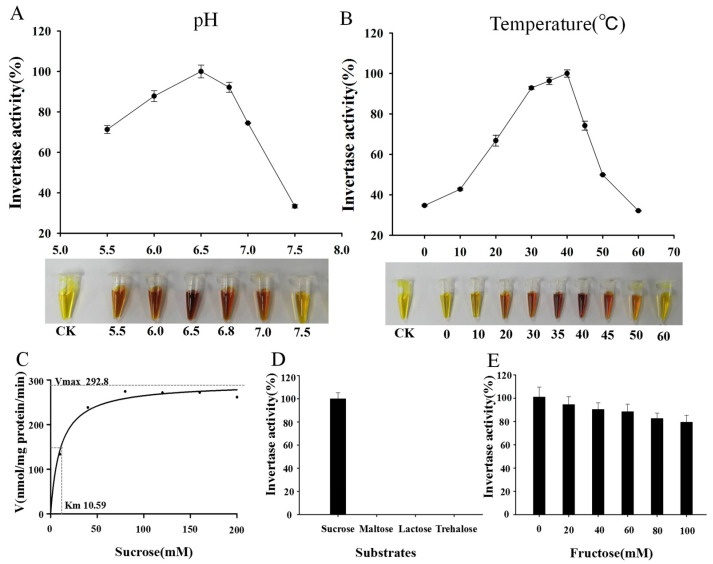
Enzymatic characteristics of MeNINV1. (**A**) The optimal pH of MeNINV1 determined through the DNS method. (**B**) The optimal temperature of MeNINV1 determined through the DNS method. (**C**) The enzymatic activity of MeNINV1 in different sucrose concentrations. (**D**) The enzymatic activity of MeNINV1 in different substrates. (**E**) Inhibition of the enzymatic activity of MeNINV1 by fructose. Data are the mean ± SD of three independent assays.

The crude enzyme from SEY2102 carrying pDR195-MeNINV1 and SEY2102 carrying pDR195(as a control) were each extracted, and the enzymatic activity was measured under different conditions. MeNINV1 catalyzed the decomposition of sucrose into reducing sugars (glucose and fructose) and then reacted with 3,5-dinitrosalicylic acid (DNS) to produce a brown-colored product. The results showed that the optimal pH of MeNINV1 was approximately 6.5, indicating that MeNINV1 belonged to A/N-Inv (Figure 2A). The highest activity was at 40 °C, but MeNINV1 still had 35% enzyme activity at 0 °C (Figure 2B). The enzymatic activity gradually increased from 0 mM to 160 mM sucrose substrate but slightly decreased at 200 mM. The Km of MeNINV1 protein was 10.59 mM at pH 6.5, and the maximum reaction rate Vmax was 292.8 nmol/mg protein/min (Figure 2C). MeNINV1 only specifically catalyzed the decomposition of sucrose, not maltose, lactose, or trehalose, indicating that MeNINV1 had specificity for sucrose (Figure 2D). The addition of fructose, a decomposition product of sucrose, to the reaction solution inhibited the enzymatic activity of MeNINV1 (Figure 2E). The enzyme activity of MeNINV1 was strongly inhibited by VB_6_, Zn^2+^, and Pb^2+^ at low concentrations (Table 1). As the concentrations of EDTA, DTT, Tris, and Mg^2+^ gradually increased, the enzymatic activity of MeNINV1 also gradually decreased. In contrast, as the concentrations of Ca^2+^ and Mn^2+^ gradually increased, the enzyme activity of MeNINV1 first decreased and then slightly increased (Table 1).

### 2.3. Subcellular Localization of the MeNINV1 Protein

**Figure 3:**
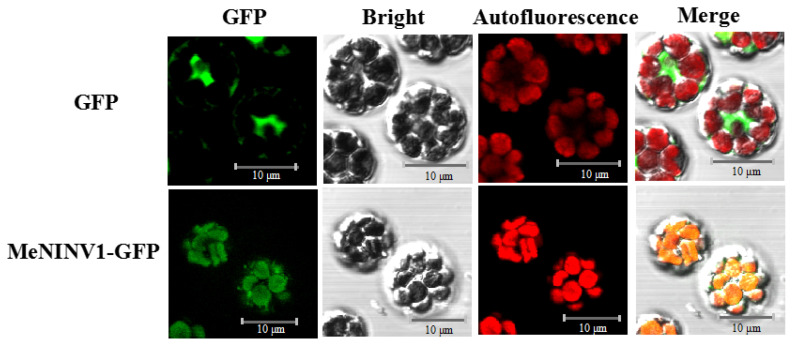
Subcellular localization of MeNINV1 in cassava mesophyll protoplasts. GFP fluorescence was observed using the green channel, and chlorophyll autofluorescence was visualized using the red channel. Bar = 10 μm.

The pCAMBIA1300-MeNINV1-GFP and pCAMBIA1300-GFP vectors were transiently transferred into cassava protoplasts to observe MeNINV1 localization under a laser confocal microscope through GFP green fluorescence. The results showed that in pCAMBIA1300-GFP-transformed cassava mesophyll cell protoplasts, GFP green fluorescence was observed in whole mesophyll cells, and the red fluorescence emitted by the chloroplasts did not overlap. In contrast, both were observed in the chloroplasts of pCAMBIA1300-MeNINV1-GFP-transformed cassava mesophyll cell protoplasts. This finding indicated that MeNINV1 was located in the chloroplasts (Figure 3).

### 2.4. The Expression of MeNINV1 in Cassava Tissues and during Tuber Root Development

**Figure 4:**
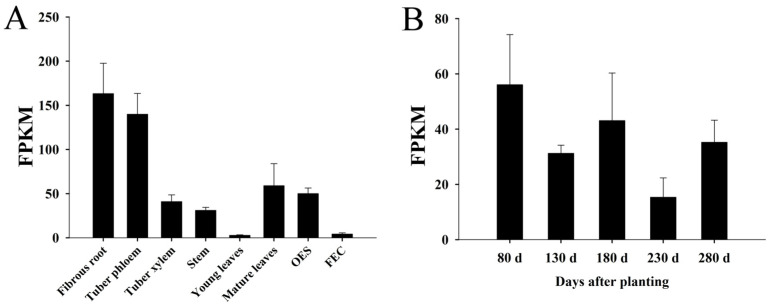
Expression analysis of the *MeNINV1* gene in cassava tissues and organs and during tuber root development. (**A**) Tissue and organ expression analysis of the *MeNINV1* gene. (**B**) Expression analysis of the *MeNINV1* gene during tuber root development. Data are the mean ± SD of three independent assays. FPKM—Fragments per kilobase per exon model per million mapped fragments.

In this study, eight tissues and organs of cassava tuber root phloem and xylem, fibrous roots, young and mature leaves, stems, fragile embryonic calli (FEC), and somatic embryos (OES), as well as three tuber root development periods at five time points during the initial stage (80 d after planting), the expanding stage (130 d and 180 d after planting), and the mature stage (230 d and 280 d after planting) were analyzed through transcriptome sequencing. The results showed that the *MeNINV1* gene was mainly expressed in fibrous roots and tuber root phloem, while its expression was relatively low in young leaves and FEC (Figure 4A). The expression levels of the *MeNINV1* gene were highest at the tuber root initial stage (80 d after planting) and showed a downward trend during tuber root growth; the lowest expression level was at 230 d after planting (Figure 4B).

### 2.5. Transformation and Identification of MeNINV1 Transgenic Arabidopsis

**Figure 5:**
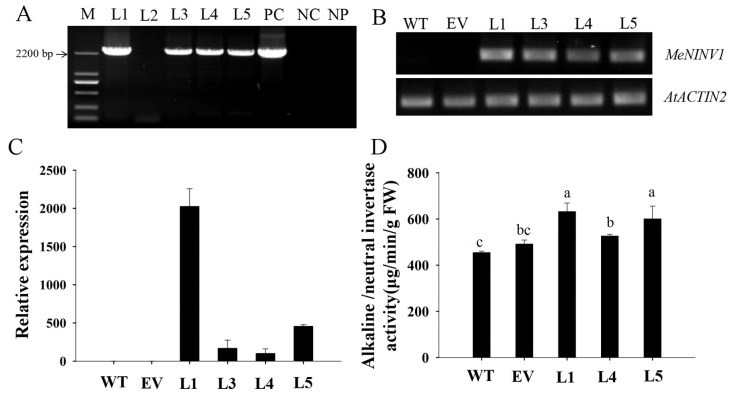
Identification of *MeNINV1* transgenic *Arabidopsis* lines. (**A**) PCR detection of T1 transgenic *Arabidopsis*, M—DL2000 marker; L1–5—transgenic plants; PC—positive control of pCAMBIA1300-MeNINV1-GFP vector; NC—negative control of WT; NP—no template PCR. (**B**) Semiquantitative RT–PCR of *MeNINV1* in T1 transgenic *Arabidopsis* lines. *AtACTIN2* was the reference gene. (**C**) The related expression levels of *MeNINV1* in T1 transgenic *Arabidopsis* lines. *AtACTIN2* was the reference gene, and the relative expression of *MeNINV1* in WT was used as a calibrator to compare with other lines for mapmaking. (**D**) A/N-Inv activity in leaves of WT and transgenic *Arabidopsis.* In (**B**–**D**): WT—wild-type *Arabidopsis thaliana* Columbia; EV—transgenic *Arabidopsis* with the pCAMBIA1300-GFP vector. Data in (**C**,**D**) are the mean ± SD of three independent assays. Significant differences were determined by one-way ANOVA followed by Duncan’s multiple range test at *p* < 0.05. Values labeled with different letters (a, b, and c) were significantly different.

*MeNINV1* fused with GFP under the control of the 35S promoter in pCAMBIA1300-MeNINV1-GFP and the pCAMBIA1300-GFP vector as control were transformed into *Arabidopsis* through the floral-dip method. After the hygromycin selection, five T1 transgenic lines were obtained, and four of them were detected with positive transformation by PCR (Figure 5A). The semiquantitative RT–PCR and qRT–PCR results showed that the *MeNINV1* gene was expressed in the four transgenic *Arabidopsis* lines, in which the L1 line had the highest expression and the L4 line had the lowest expression (Figure 5B–C). After three generations of selection on Murashige and Skoog (MS) medium containing 50 mg/L hygromycin, the homozygous transgenic lines L1, L4, and L5 were obtained to further study *MeNINV1* gene function in plants (Figure S1). The A/N-Inv activities in the three *MeNINV1* transgenic plants were higher than those in the control vector and wild type (WT) plants, and the L1 and L5 had higher A/N-Inv activities, which was consistent with the *MeNINV1* expression levels in the transgenic *Arabidopsis* (Figure 5D).

### 2.6. Overexpression of MeNINV1 in Arabidopsis Promoted Plant Growth and Delayed Flowering Time

**Figure 6:**
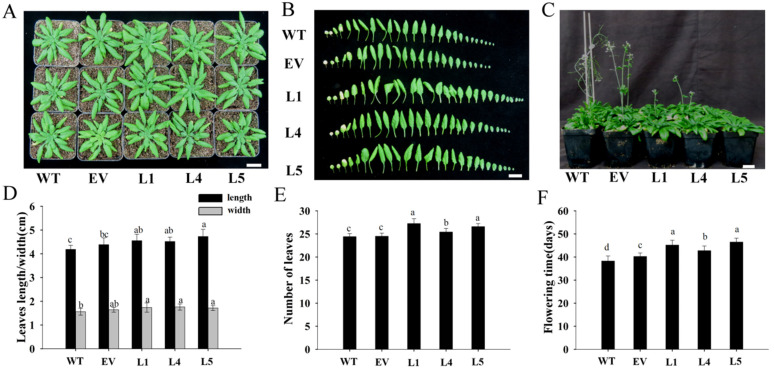
Phenotypes of *MeNINV1* transgenic *Arabidopsis* and their controls. (**A**) Phenotypes of plants. (**B**) Morphology of leaves per plant. (**C**) Flowering initials. (**D**) The length and width of the longest leaves. (**E**) The numbers per plant. (**F**) Flowering time of the plants. L1, L4, and L5 are *MeNINV1* transgenic *Arabidopsis* lines. EV is a pCAMBIA1300-GFP transgenic *Arabidopsis* line. WT is wild-type *Arabidopsis*. Plants in (**A**,**B**,**D**,**E**) are five weeks old; (**C**,**F**) are flowing initials. Data in (**D**–**F**) are the means of 20 leaves (**D**) or plants (**E**,**F**) with three independent assays. Significant differences were determined by one-way ANOVA followed by Duncan’s multiple range test at *p* < 0.05. Values labeled with different letters (a, b, and c) were significantly different. Bar in B = 2 cm.

The *MeNINV1* homozygous lines L1, L4, and L5, EV transgenic *Arabidopsis*; and WT *Arabidopsis* were cultured under the same conditions (Figure 6A). The leaf morphologies of *MeNINV1* transgenic *Arabidopsis* were not significantly different from those of EV and WT (Figure 6B). Still, the leaf length and width were significantly larger than WT (Figure 6D). The numbers of leaves per plant were also significantly greater than those of EV and WT (Figure 6E). The flowering time of *MeNINV1* transgenic *Arabidopsis* was significantly later than that of EV and WT, which was delayed by 3 to 6 d (Figure 6C,F). However, there were no significant differences in the morphologies of the flowers, pods, and seeds among the *MeNINV1* transgenic *Arabidopsis* and EV and WT (Figure S2). These results indicate that the *MeNINV1* gene promotes the growth of *Arabidopsis* and delays plant flowering.

### 2.7. Overexpression of MeNINV1 in Arabidopsis Increased Sucrose Metabolism and Starch Synthesis

**Figure 7:**
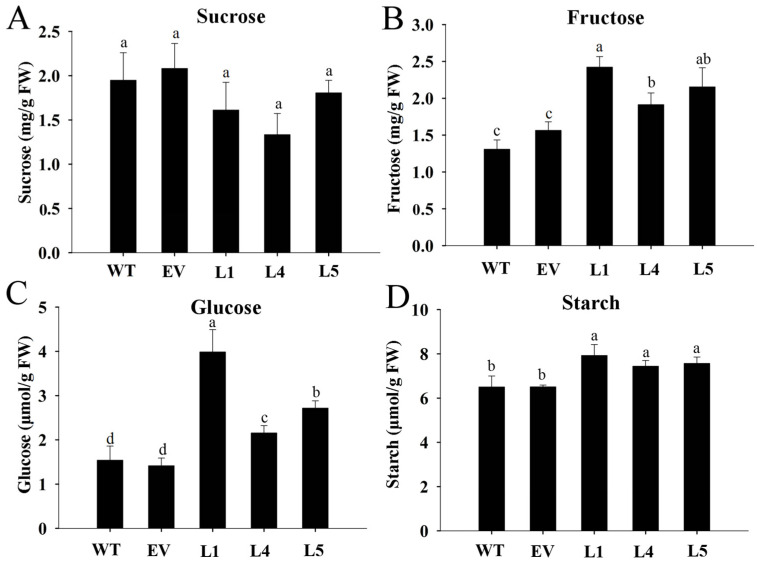
Analysis of sucrose, fructose, glucose, and starch in *MeNINV1* transgenic *Arabidopsis* and their controls. (**A**) Sucrose content. (**B**) Fructose content. (**C**) Glucose content. (**D**) Starch content. The plants used for analysis were six weeks old. L1, L4, and L5 are *MeNINV1* transgenic *Arabidopsis* lines. EV is a pCAMBIA1300-GFP transgenic *Arabidopsis* line. WT is wild-type *Arabidopsis*. Data are the mean ± SD of three independent assays. Significant differences were determined by one-way ANOVA followed by Duncan’s multiple range test at *p* < 0.05. Values labeled with different letters (a, b, and c) were significantly different.

To explore whether MeNINV1 was involved in sugar metabolism in transgenic *Arabidopsis,* the contents of sucrose, fructose, glucose, and starch were detected. The results showed that the sucrose content in the *MeNINV1* transgenic *Arabidopsis* lines decreased slightly but was not significantly different from that in WT and EV (Figure 7A). In contrast, the fructose, glucose, and starch contents increased significantly (Figure 7B–D). This indicated that the increased A/N-Inv activity in *MeNINV1* transgenic *Arabidopsis* increased the decomposition of sucrose to produce more glucose and fructose, leading to the increased synthesis of starch.

### 2.8. No Change in the Overexpression of MeNINV1 in Arabidopsis under Abiotic Stress

Sucrose metabolism has often been associated with the abiotic stress resistance of plants. In this study, several stressors, such as salt, drought, and cold, were tested in *MeNINV1* transgenic *Arabidopsis*. The results showed that the *MeNINV1* transgenic *Arabidopsis* did not have enhanced tolerance to stress (Figure S3).

## 3. Discussion

The yeast-restoration experiment identified that MeNINV1 could restore the invertase function in the invertase mutant strain SEY2102 (Figure 1), which was the same as Ta-A/N-Inv1 from wheat [25]. Further studies have found that MeNINV1 only catalyzed the decomposition of sucrose, not maltose, trehalose, and lactose (Figure 2D). Many studies have found that A/N-Inv could only decompose sucrose, such as At-CYT-INV1 from *Arabidopsis* [19] and INV from carrot [26]. The optimal enzymatic pH of A/N-Inv has been determined to be 6.5–8.0 and that of A-Inv 4.5–5.0 [9]. The optimal pH for MeNINV1 to catalyze sucrose decomposition was 6.5, which was the same as A/N-InvC from *Arabidopsis* [27]. Generally, the optimal reaction temperature of A/N-Inv has been 37–45 °C [7,11], but high catalytic activity for sucrose degradation at low temperatures was also found in the alkaline invertase rInvHJ14 from *Bacillus* [28]. The optimal reaction temperature of MeNINV1 was 40 °C, but its enzyme activity remained at approximately 35% at a low temperature (0 °C). The kinetic curve of MeNINV1 was 10.59 mM at pH 6.5, which was within the range (Km = 10–30 mM) of the A/N-Inv from *Arabidopsis*, as had been reported previously [29]. In enzymatic reactions, substrates can inhibit the activity of the enzyme. In this study, increased fructose in the reaction solution decreased the enzymatic activity of MeNINV1, which indicates that fructose could be one of the products. This phenomenon was also found in alkaline invertase from rice [30] and HbNIN2 from rubber [11]. The enzyme activity of MeNINV1 was strongly inhibited by VB6, Zn^2+^, and Pb^2+^ at low concentrations, and other metal ions and chemicals (e.g., EDTA, DTT, Tris, and Mg^2+^) inhibited its activity, which was similar to the reported A/N-Inv from carrots [27], *Arabidopsis* [28], and rice [29], except that a low concentration of vitamin B6 increased the enzymatic activity of HbNIN2 [11]. These studies on the enzymatic properties of A/N-Inv were similar, indicating that A/N-Inv has been functionally conserved among different species.

A/N-Inv was divided into α-group and β-group according to the evolutionary relationships [24]. Studies have reported that all β-group A/N-Inv have been located in the cytoplasm [31]. The α A/N-Inv group was divided into classes α1 and α2, and most of class α1 were located in the mitochondria or the chloroplasts [19,32,33], and class α2 were located in plastids [18]. MeNINV1 was classified in the α1 group, along with PtNIN1 and At3G06500 (A/N-InvC). PtNIN1 was shown to localize in the chloroplasts and the mitochondria [20], and A/N-InvC was shown to localize in the mitochondria [19]. The online software Cell-PLoc-2 [34] and WoLF PSORT [35] were used in this study to predict the possible localization of MeNINV1 in the chloroplasts. Subsequently, MeNINV1-GFP was transiently expressed in cassava protoplasts, and MeNINV1 was located in the chloroplasts. The localization of MeNINV1 was different from that of PtNIN1 and A/N-InvC, which could have been caused by differences in protein sequences.

The determination of the invertase activity at each stage of cassava tuber root starch accumulation identified that A/N-Inv had the highest activity in tubers, indicating that it played an important role in this process [24,36,37]. The A/N-Inv activity of high-starch cassava tuber varieties was significantly higher than that of low-starch varieties [23]. Tissue-specific analysis showed that the *MeNINV1* gene was mainly expressed in the fibrous roots and the tuber root phloem, which was different from the previous study where *MeNINV1* was mainly expressed in the stems [24], possibly due to the different growth states of cassava (the previous cassava was planted for 80 d). The expression level of *MeNINV1* was the highest at 80 d after planting and the lowest at 230 d after planting, which was accompanied by the accumulation of starch during tuber root development, indicating that *MeNINV1* might be a key gene in this process, which was consistent with previous research [24].

*MeNINV1* transgenic *Arabidopsis* increased plant biomass and delayed flowering, suggesting that the *MeNINV1* gene promoted the vegetative growth of *Arabidopsis* and delayed reproductive growth (Figure 6). A/N-Inv plays an important role in the catabolism of sucrose, and the decomposition products of sucrose are crucial for plant growth and development [38]. Many reports have pointed out that invertase has affected plant growth and development. For example, the *Arabidopsis* A/N-InvG affected the development of lateral roots [39]. The mutations in the *OsCYT-INV1* gene led to reduced A/N-Inv activity and an inhibited rice root cell development and reproductivity [40]. The homozygous mutants of *LjINV1* showed a severe reduction in the growth of roots and shoots, a change in cellular development, and impaired flowering in *Lotus japonicus* [41]. The mutation of *Arabidopsis* A/N-InvC affected the growth and development of plants, especially during the germination and flowering stages [19]. The expression of the *CIN12* gene in *Populus tomentosa* was disturbed, and the activity of cytoplasmic invertase was inhibited by 38–55%, resulting in a 9–13% reduction in crystalline cellulose, which affected the formation of wood [31]. The expression of *SlCIN7* in tomatoes was inhibited, resulting in reduced pollen vigor and hindered pollen germination [21]. These studies fully demonstrate that invertase controlled plant growth and development by affecting sugar signaling.

Previous studies have indicated that some A/N-Invs might be involved in sucrose catabolism and starch accumulation. The alkaline/neutral invertase PtrA/NINV of *Poncirus trifoliata* was heterologously expressed in tobacco, and the A/N-Inv activity of transgenic tobacco was increased. There was no change in the sucrose content under normal conditions, but the sucrose content was significantly decreased, and the reduced sugar content was significantly increased under stress [20]. The double mutations in the alkaline/neutral invertase genes *A/N-InvG* and *A/N-InvI* in *Arabidopsis* reduced the activity of A/N-Inv by 40%, and there was no starch accumulation in the root-cap cells [15]. In *MeNINV1* transgenic *Arabidopsis*, the A/N-Inv activity in the leaves was increased, and the contents of fructose, glucose, and starch were significantly increased, although the sucrose content was not significantly changed. This result suggested that the *MeNINV1* gene affected sucrose catabolism and promoted starch synthesis due to increased glucose and fructose. The lack of change in sucrose content in the leaves of *MeNINV1* transgenic *Arabidopsis* could have been caused by photosynthesis.

Studies have also found that some A/N-Invs responded to abiotic stress in plants by affecting sucrose metabolism [20,38]. However, *MeNINV1* transgenic *Arabidopsis* did not significantly improve salt, drought, or low-temperature resistance. This finding indicated that the *MeNINV1* gene does not participate in the stress response in plants.

## 4. Materials and Methods

### 4.1. Strains and Plasmids

The *Escherichia coli* strain DH5α and the *Agrobacterium tumefaciens* strain GV3101 used to construct the recombinant vectors and for the transformation of *Arabidopsis* were stored in our laboratory. The invertase mutant yeast strain SEY2102 used for the yeast functional complementation experiment was kindly provided by Jie Liu (Northwest A&F University, Xianyang, China). A yeast shuttle vector pDR195 used for MeNINV1 protein(NCBI accession: AEP31948.1) expression, the binary vector pCAMBIA1300-35S-GFP used for *MeNINV1* subcellular localization, and the *Arabidopsis thaliana* (columbia-0) used for transformation were stored in our laboratory.

### 4.2. Functional Analysis of MeNINV1 by Yeast Complementation

The full-length open reading frame (ORF) of the *MeNINV1* (NCBI accession: JN616390.1) was amplified by PCR with the pDR195-M1-F/R primers containing *Not* I and *Sal* I restriction sites (Table S1). The amplified product and the pDR195 vector were double-digested with restriction enzymes *Not* I and *Sal* I and then ligated by using T4 ligase to form the recombinant plasmid pDR195-MeNINV1. The pDR195 vector and pDR195-MeNINV1 were transformed into the yeast mutant SEY2102 according to the lithium acetate (LiAc) transformation method [42].

SEY2102 yeast cells containing pDR195 or pDR195-MeNINV1 were grown on select SD media (lacking uracil) containing 2% of either sucrose or fructose as a sole carbon source for 3 d at 30 °C to observe their growth.

### 4.3. Analysis of the Enzymatic Characteristics of MeNINV1

Yeast cells containing the pDR195 or pDR195-MeNINV1 vectors were cultured in a YPD solution medium for 2 d at 30 °C. The cells were collected by centrifugation at 12,000 rpm and 4 °C for 10 min and washed twice with PBS buffer. The collected yeast cells were ground using liquid nitrogen and then transferred to PBS buffer (pH = 6.5) and centrifuged at 12,000 rpm at 4 °C for 10 min. The supernatant was a crude enzyme solution. The concentration of the extracted protein was determined by the Bradford method [43]. Alkaline/neutral invertase activity was measured using the DNS method [44], the activity using crude serum from SEY2102 carrying pDR195 as essential control. To determine the optimal reaction conditions, different pH values, temperatures, and sucrose concentrations were tested. Under the optimal conditions, the effects of different substrates, fructose concentrations, metal ions (Ca^2+^, Mg^2+^, Mn^2+^, Zn^2+^, and Pd^2+^), and chemical reagents (Tris, EDTA, DTT, and VB6) on the enzyme activity were studied.

### 4.4. Plant Materials

The cassava (*Manihot esculenta*, South China 8 (SC8)) used in this study was planted in Lingao, Hainan, China. The cassava tuber roots were collected at 80, 130, 180, 230, and 280 d after planting for RNA-seq. Fragile embryogenic calli and somatic embryos on the medium were chosen for RNA-seq. The tuber phloem, tuber xylem, fibrous roots, tender leaves, mature leaves, and cassava stems were collected at 180 d after planting for RNA-seq.

In vitro plantlets of cassava SC8 used for cassava mesophyll protoplast isolation were kept in MS medium (supplemented with 2% sucrose and 0.8% agarose, pH 5.8) at 28 °C under 12 h/8 h (light/darkness) for 6–10 weeks to obtain fully expanded leaves.

*A. thaliana* Col-0 was used as the wild-type (WT) control in this study, and all transgenic *Arabidopsis* lines were generated on a background of Col-0. Seeds were sterilized with 75% (*w*/*v*) alcohol for 10 min, washed 1 time with absolute ethanol, transferred to sterile filter paper with a pipette, and air-dried for approximately 30 min until the alcohol had completely evaporated. The sterilized seeds were sown on 1/2 MS medium (2% (*w*/*v*) sucrose, 0.7% (*w*/*v*) agar) and vernalized at 4 °C for 3 d in the dark. Then the seeds were cultured under the conditions of 22 °C, 110 µmol/m^2^/s light intensity, 16 h light/8 h dark, and 70% relative humidity for 10 d. Finally, the seedlings were transferred to pots filled with a mixture of vermiculite and soil, and they were watered with a nutrient solution every 3 d. The plants were grown under the same conditions.

### 4.5. Expression Analysis of the MeNINV1 Gene in Cassava

The expression levels of *MeNINV1* in different tissues and organs, as well as tuber root development periods, were calculated according to the SC8 cassava RNA sequencing data (unpublished).

### 4.6. Subcellular Localization of the MeNINV1 Protein

The full-length ORF of *MeNINV1* without a stop codon was amplified by PCR with the 1300-M1-F/r primers containing *Kpn* I and *Sal* I restriction sites (Table S1). The amplified product and the pCAMBIA1300-GFP vector were double-digested with the restriction enzymes *Kpn* I and *Sal* I and then ligated using T4 ligase to form the recombinant plasmid pCAMBIA1300-MeNINV1-GFP. The cassava mesophyll protoplast isolation and transformation methods were described by Wu et al. [45]. The GFP fluorescence (green channel, 488 nm) and chlorophyll autofluorescence (red channel, 568 nm) were observed under a laser confocal microscope (OLYMPUS FV1000, Olympus, Tokyo, Japan).

### 4.7. Transformation and Identification of MeNINV1 Arabidopsis

*Agrobacterium* GV3101 with pCAMBIA1300-MeNINV1-GFP and pCAMBIA1300- GFP were transformed into *Arabidopsis* Col-0 by the flower-infusion method. The infected mature seeds (T0) were collected and cultured on 1/2 MS (containing 50 mg/L hygromycin) solid medium to screen for positive transformants. *Arabidopsis* seedlings rooting on hygromycin medium were identified by leaf PCR with 2 × M5 HiPer Superluminal mix-with blue dye (Mei5bio, Beijing, China) using the 1300-F/R primers (Table S1). The positive transgenic lines (T1) were generated and examined by semiquantitative RT–PCR, and qPCR. *AtActin2* (GenBank Accession: NM_112764) was used as an internal control. pCAMBIA1300-GFP-transformed *Arabidopsis* was used as empty vector control. Wild-type (WT) *Arabidopsis* Col-0 was used as a negative control.

The positive transgenic line (T1) seeds were collected and cultured on 1/2 MS (containing 50 mg/L hygromycin) solid medium to screen for single-copy lines. Seeds of the single-copy transgenic lines (T2) were collected and cultured on 1/2 MS (containing 50 mg/L hygromycin) solid medium to screen for homozygous lines. For further experiments, T3 homozygous *Arabidopsis* transgenic lines (L1, L4, and L5) were used.

When the *Arabidopsis* plants were five weeks old, the leaves of pCAMBIA1300-MeNINV1-GFP, pCAMBIA1300-GFP T3 transgenic lines, and WT *Arabidopsis* were analyzed for alkaline/neutral invertase activity using an A/N-Inv kit (Keming, Suzhou, China).

### 4.8. Phenotypic Statistics of Transgenic Arabidopsis

The seeds of pCAMBIA1300-MeNINV1-GFP, pCAMBIA1300-GFP T3 transgenic lines, and WT *Arabidopsis* were sown in vermiculite. When the plants had grown four true leaves, the seedlings with similar growth were transferred to plastic pots (7 × 7 cm). After 5 weeks under the conditions of 22 °C, 110 µmol/m^2^/s light intensity, 16 h light/8 h dark, and 70% relative humidity, the morphological data of plants, including leaf length/width, number of leaves, and flowering time, were calculated.

### 4.9. Detection of Various Sugars in Transgenic Arabidopsis

The leaves of 6-week-old pCAMBIA1300-MeNINV1-GFP and pCAMBIA1300-GFP transgenic lines as well as WT *Arabidopsis* were collected, and the contents of sucrose, glucose, fructose, and starch were determined. The above indicators were measured using sucrose, glucose, fructose, and starch kits (Keming, Suzhou, China).

### 4.10. Analysis of Abiotic Stress in Transgenic Arabidopsis

Ten-day-old seedlings (L1, L4, L5, EV, and WT) were transferred to 1/2 MS medium supplemented with 0, 100, 200, or 300 mM mannitol or 0, 50, 100, or 150 mM NaCl under the same conditions for 10 d as the drought- and salt-stress treatments. Ten-day-old seedlings (L1, L4, L5, EV, and WT) were transferred to 1/2 MS medium at 4 °C for 10 d as a cold-stress treatment.

### 4.11. Statistical Analysis

The data are presented as the mean ± SD and were compared using the Statistical Product and Service Solutions (SPSS, IBM, Armonk, NY, USA) software with one-way analysis of variance (ANOVA) followed by Duncan’s multiple range test at the significance level of *p* < 0.05.

## 5. Conclusions

In this study, MeNINV1 was confirmed to function as invertase to catalyze sucrose decomposition in yeast. The enzymatic properties of MeNINV1 were an optimal pH of 6.5, a reaction temperature of 40 °C, and sucrose as its specific catalytic substrate. Chemical reagents, metal ions, and fructose can inhibit A/N-INV enzymic activity. In cassava, the *MeNINV1* gene was mainly expressed in the fibrous roots and the tuber root phloem, and its expression decreased as the tuber root grew. MeNINV1 was confirmed as located in the chloroplasts. *MeNINV1*-overexpressing *Arabidopsis* had a higher A/N-INV activity, and the increased glucose, fructose, and starch content in the leaves promoted plant growth and delayed flowering time but did not change their resistance to abiotic stress.

## Figures and Tables

**Table 1:** Effects of metal ions and chemicals on the activity of MeNINV1.

Chemicals	Concentration (mM)	Relative Activity (%)	Metal Ions	Concentration (mM)	Relative Activity (%)
None	-	100 ± 6.54	Ca^2+^	20	81.22 ± 0.47
Tris	1	90.32 ± 7.84		40	33.25 ± 0.55
	2	84.85 ± 4.15		60	22.29 ± 0.57
	4	75.53 ± 3.23		80	43.85 ± 0.12
	6	69.33 ± 3.52		100	41.29 ± 0.46
	8	62.44 ± 1.97	Mg^2+^	20	67.54 ± 0.32
	10	56.15 ± 2.72		40	52.39 ± 1.66
	20	41.72 ± 2.32		60	40.87 ± 0.19
	50	30.70 ± 0.56		80	32.50 ± 0.63
	100	30.11 ± 0.13		100	24.18 ± 0.56
EDTA	20	52.28 ± 0.28	Mn^2+^	20	46.63 ± 0.14
	40	46.06 ± 0.38		40	18.86 ± 0.28
	60	31.20 ± 0.41		60	27.91 ± 0.48
	80	19.18 ± 0.13		80	31.54 ± 0.36
	100	15.46 ± 0.16		100	38.93 ± 0.17
DTT	20	80.71 ± 1.99	Zn^2+^	20	44.60 ± 0.14
	40	68.43 ± 0.21		40	15.77 ± 0.25
	60	33.80 ± 0.10		60	12.97 ± 0.05
	80	25.17 ± 0.21		80	12.95 ± 0.04
	100	25.34 ± 0.44		100	12.83 ± 0.14
VB6	20	24.83 ± 0.13	Pb^2+^	20	48.64 ± 0.11
	40	15.20 ± 0.09		40	13.28 ± 0.02
	60	14.17 ± 0.08		60	13.28 ± 0.13
	80	15.97 ± 0.11		80	13.63 ± 0.09
	100	16.59 ± 0.30		100	13.36 ± 0.06

Tris: Tris (hydroxymethyl) methyl aminomethane THAM; EDTA: ethylenediaminetetraacetic acid; DTT: dithiothreitol; VB6: Vitamin B6. Data are the mean ± SD of three independent assays.

## Supplementary Materials

The following supporting information can be downloaded at: https://www.mdpi.com/article/10.3390/plants11070946/s1, Figure S1: Screening of single-copy and homozygous transgenic *Arabidopsis*, Figure S2: The floral organs and fruit pods of *MeNINV1*-overexpressing *Arabidopsis* lines, Figure S3: Resistance analysis of *MeNINV1*-overexpressing *Arabidopsis* lines, Table S1: The primers used in the study.
