# Supplementary material for: MeNINV1: An Alkaline/Neutral Invertase Gene of Manihot esculenta, Enhanced Sucrose Catabolism and Promoted Plant Vegetative Growth in Transgenic Arabidopsis
Source: Plants (Basel). 2022 Mar 31;11(7):946. doi: 10.3390/plants11070946 (PMC9003190; doi:10.3390/plants11070946)
Supplement: Supplementary file 1 [file plants-11-00946-s001.zip › plants-1616565-supplementary.pdf]

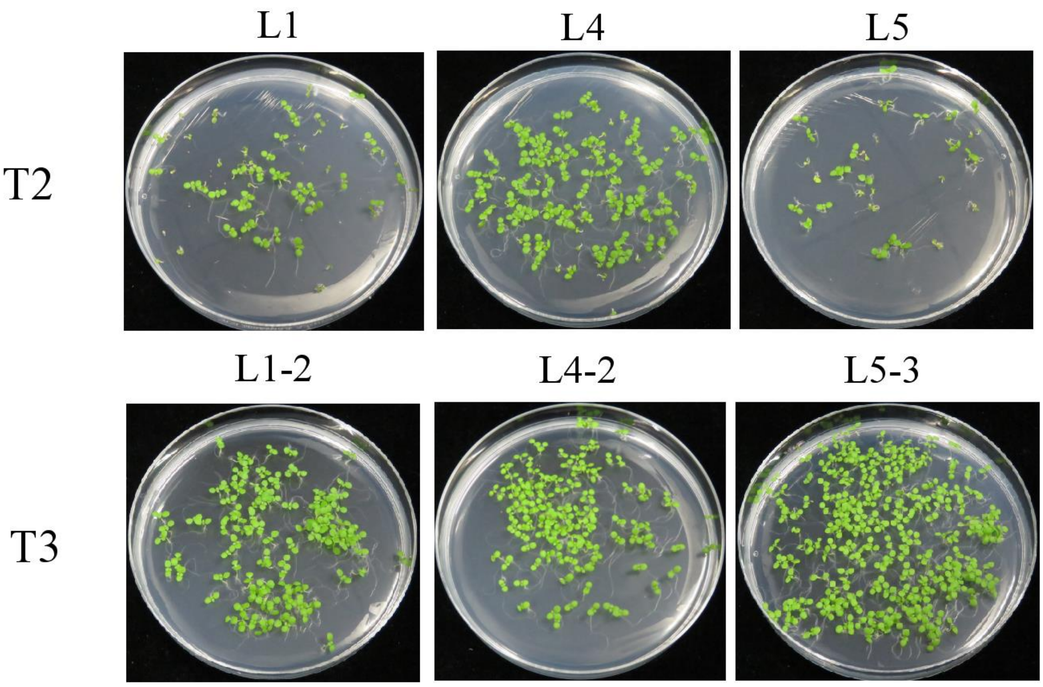

Figure S1 Screening of single-copy and homozygous transgenic *Arabidopsis*.

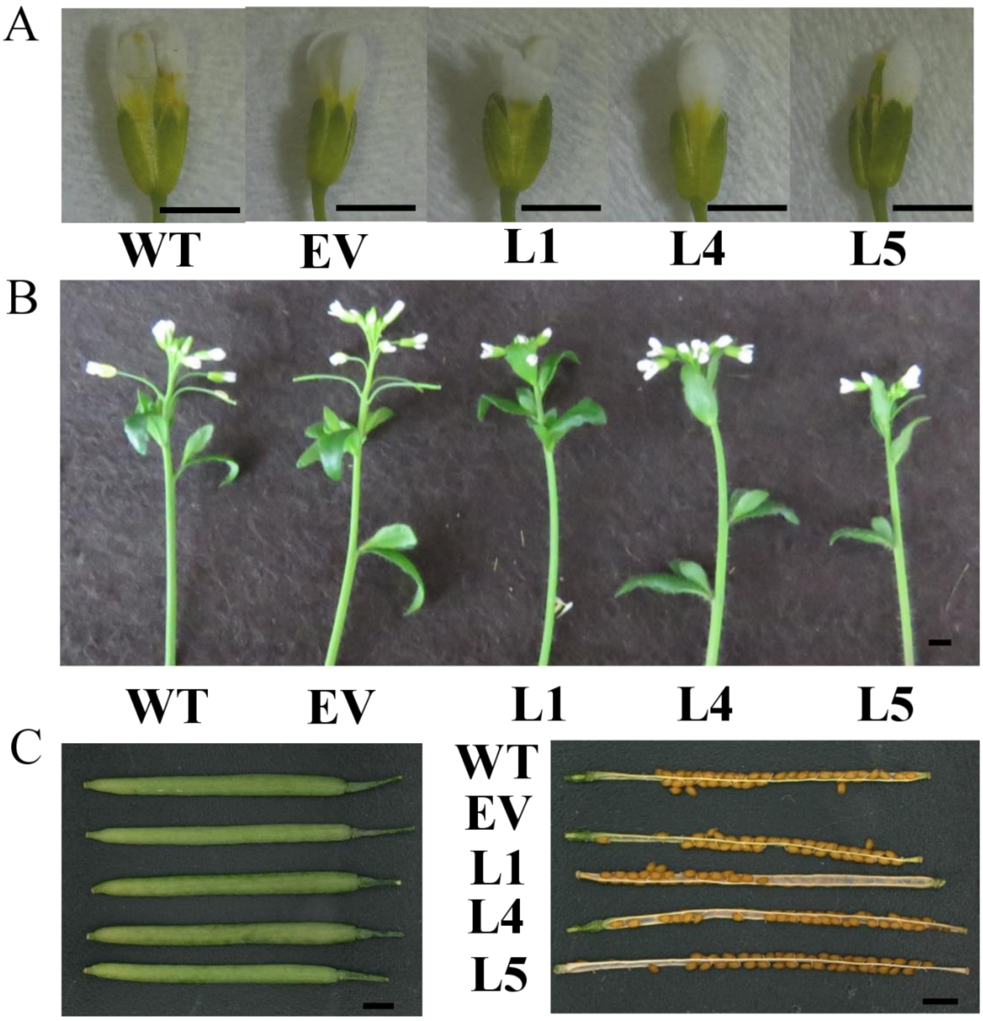

Figure S2 The floral organs and fruit pods of *MeNINV1*-overexpressing *Arabidopsis* lines. (A) Flower, (B) inflorescence, and (C) fruit pods and seeds. L1, L4, and L5 are *MeNINV1* transgenic *Arabidopsis* lines. EV is a pCAMBIA1300-GFP transgenic *Arabidopsis* line. WT is wild-type *Arabidopsis*. Bar = 0.5 cm.

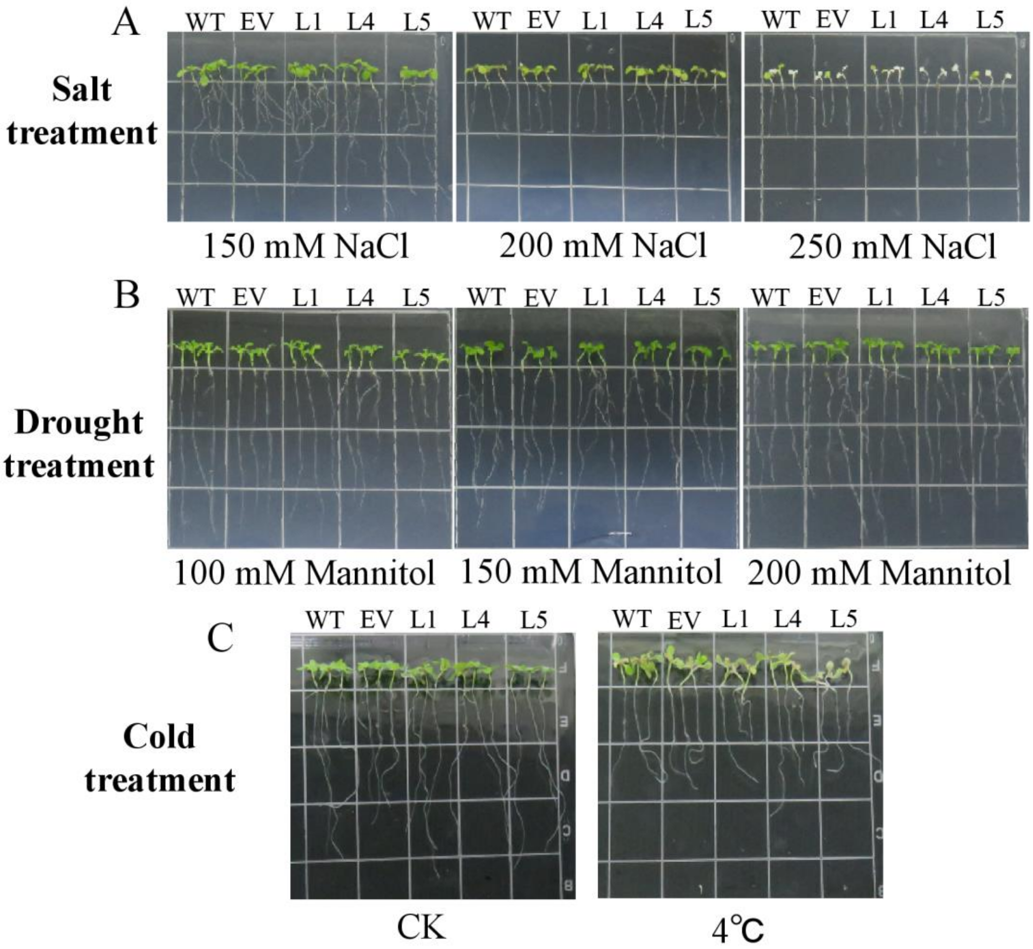

Figure S3 Resistance analysis of *MeNINV1*-overexpressing *Arabidopsis* lines. (A) Salt stress analysis. (B) Drought stress analysis. (C) Cold stress analysis. L1, L4, and L5 are *MeNINV1* transgenic *Arabidopsis* lines. EV is a pCAMBIA1300-GFP transgenic *Arabidopsis* line. WT is wild-type *Arabidopsis*.

Table S1 The primers used in the study

Name	Sequence 5'-3'
pDR195-M1-F	TATGCGGCCGCATGAATACTAGTAGTTG(*Not* I)
pDR195-M1-R	ATAGTCGACTCAGGCTTGCTTCTGG(*Sal* I)
1300-M1-F	TATGGTACCGGCTTGCTTCTGGGATC(*Kpn* I)
1300-M1-R	CGCGTCGACATGAATACTAGTAGTTGTATTGTA(*Sal* I)
pDR195-F	CGCCCAATACGCAAACCG
pDR195-R	GATGTGCTGCAAGGCGATTAA
1300-F	GTTGATACATATGCCCGTCG
1300-R	CTCGCCCTTGCTCACCAT
MeNINV1-qPCR-F	TTTGTTACCCTGCTTTGGAAC
MeNINV1-qPCR-R	AGAAACTGCCCTCTGTGCTAA
AtACTIN2-qPCR-F	CGTTTGTGGGAATGGAAGCT
AtACTIN2-qPCR-R	TTGCTCATACGGTCAGCGATA
